# Empirical study on psychosocial factors in liver cancer treatment: implications for medical student education in shared decision-making

**DOI:** 10.3389/fpubh.2026.1831463

**Published:** 2026-06-29

**Authors:** Minling Cao, Ming Lin, Yanmei Deng, Junmin Jiang

**Affiliations:** Department of Hepatology, Guangdong Provincial Key Laboratory of Clinical Research on Traditional Chinese Medicine Syndrome, The Second Affiliated Hospital of Guangzhou University of Chinese Medicine, Guangzhou, China

**Keywords:** guideline adherence, hepatocellular carcinoma, psychosocial factors, shared decision-making, treatment decision-making

## Abstract

**Objective:**

To assess the impact of psychosocial factors on hepatocellular carcinoma (HCC) treatment decisions, guideline adherence, and patient survival, and to derive empirical insights for shared decision-making (SDM) training in medical education.

**Methods:**

In this single-center retrospective cohort study, we analyzed data from 1,928 patients with primary HCC. We collected baseline characteristics, psychosocial factors (payment method, psychological anxiety), treatment choices, and overall survival. Guideline adherence was determined based on the BCLC staging system.

**Results:**

Payment method and psychological anxiety were independent predictors of both treatment selection and guideline adherence (*p* < 0.05). Overall, only 49.95% of treatment decisions were guideline-adherent. Notably, while guideline-adherent management was associated with better overall survival (*p* = 0.02), a critical finding emerged in patients with intermediate-stage (BCLC-B) disease. In this subgroup, those treated contrary to guidelines demonstrated a significant survival advantage (*p* < 0.05).

**Conclusion:**

Psychosocial factors are significant drivers of deviation from clinical guidelines in HCC care, creating a complex relationship with survival outcomes. The paradoxical survival benefit observed in non-adherent BCLC-B patients underscores the limitations of a one-size-fits-all guideline approach and highlights the necessity for individualized, patient-centered care. These findings provide a strong empirical foundation for integrating SDM and psychosocial assessment into medical curricula.

## 1 Introduction

Hepatocellular carcinoma (HCC) is a common malignancy in China with a relatively poor prognosis (1). Its treatment strategy is highly complex, requiring strict adherence to objective indicators such as tumor stage and liver function, and reference to internationally recognized clinical practice guidelines (e.g., the BCLC staging treatment strategy) (2). However, in real-world clinical settings, treatment decisions are not purely biomedical processes but are profoundly influenced by various non-clinical factors such as the patient’s psychological state, socioeconomic background, and doctor-patient communication (3). This influence often leads to deviations between the final treatment plan and guideline recommendations.

Shared decision-making (SDM) is a modern medical communication model that emphasizes the participation of both doctors and patients (4). It combines the best clinical evidence with the patient’s values and preferences to formulate the most suitable treatment plan for the individual. This is crucial for HCC treatment, which involves multiple options and complex trade-offs. Currently, medical education in China still focuses on disease knowledge and clinical skills, with insufficient training on how to assess and integrate psychosocial factors and how to effectively implement SDM (5).

This study aims to empirically explore the impact of psychosocial factors (e.g., payment method, anxiety state) on treatment decisions and their adherence to guidelines through a retrospective analysis of clinical data from a large cohort of liver cancer patients. It also analyzes the actual effect of different decisions on patient survival outcomes. The research findings are intended to provide data support for a “patient-centered” clinical mindset and offer empirical evidence and teaching entry points for effective shared decision-making instruction in medical students.

## 2 Methods

### 2.1 Study design and participants

This was a single-center retrospective cohort study. A total of 1928 patients with hepatocellular carcinoma who received initial treatment at our hospital between January 2015 and December 2020 were consecutively enrolled. Inclusion criteria consisted of: diagnosis of HCC confirmed by pathology or clinical imaging criteria, and availability of complete clinical and follow-up data. Exclusion criteria were: presence of other malignant tumors, initial treatment performed at another hospital, or incomplete data.

### 2.2 Data collection

*Baseline data*: age, gender, body mass index (BMI), hepatitis B virus (HBV) infection status, Child-Pugh grade, BCLC stage, presence of metabolic diseases.

*Psychosocial factors*: medical insurance payment method (categorized as urban employee insurance, government-funded, new rural cooperative medical scheme, or completely out-of-pocket). Psychological anxiety was assessed by reviewing medical records for documented clinical diagnoses of anxiety at the time of admission. All hospitalized HCC patients underwent psychological evaluation using the validated Hospital Anxiety and Depression Scale-Anxiety subscale (HADS-A).

*Treatment data*: the primary treatment received by the patient, categorized as open hepatectomy, laparoscopic hepatectomy, local ablation therapy, or transarterial chemoembolization (TACE).

*Outcome indicator*: overall survival (OS), defined as the time from the date of first treatment to the date of death or last follow-up.

### 2.3 Group definitions

*Guideline adherence assessment*: guideline adherence was assessed based on the Barcelona Clinic Liver Cancer (BCLC) staging system guidelines prevailing during the study period. Two senior attending physicians, blinded to patient outcomes, independently judged whether the initial treatment decision adhered to the BCLC recommendations according to the following criteria: BCLC stage 0 (very early stage): Recommended treatment = ablation or resection; adherence = receipt of either. BCLC stage A (early stage): Recommended treatment = resection, ablation, or transplantation; adherence = receipt of any of these. BCLC stage B (intermediate stage): Recommended treatment = transarterial chemoembolization (TACE); adherence = receipt of TACE as first-line therapy.

*BCLC stage C (advanced stage)*: recommended treatment = systemic therapy (e.g., sorafenib); adherence = receipt of systemic therapy. BCLC stage D (terminal stage): Recommended treatment = best supportive care; adherence = receipt of supportive care without active anti-tumor treatment. Any deviation from these stage-specific recommendations was classified as “non-guideline-adherent.” Disagreements between the two reviewers were resolved by discussion or adjudication by a third chief physician.

*Treatment grouping*: patients were divided into four groups based on the actual treatment received—Open Hepatectomy, Laparoscopic Hepatectomy, Ablation, TACE—for baseline characteristic comparisons.

### 2.4 Statistical analysis

Statistical analysis was performed using SPSS software (version 26.0). Measurement data were expressed as mean ± standard deviation, and intergroup comparisons were conducted using analysis of variance. Count data were expressed as number (percentage), and intergroup comparisons were made using the chi-square test. The cox proportional hazards regression model was used for multivariate survival analysis, calculating hazard ratios (HR) and their 95% confidence intervals (CI). Survival curves were plotted using the Kaplan–Meier method, and differences in survival between groups were compared using the Log-rank test. A *p*-value < 0.05 was considered statistically significant.

## 3 Results

### 3.1 Patient baseline characteristics and treatment choice differences

This study included 1928 HCC patients with a mean age of 57.96 ± 14.78 years, predominantly male (82.36%). As shown in Table 1, there were significant differences (*p* < 0.05) among the different treatment groups (open surgery, laparoscopic surgery, ablation, TACE) in terms of age, gender, payment method, psychological anxiety, metabolic diseases, Child-Pugh grade, and BCLC stage. Notably, the distribution of payment method showed extremely significant differences (*p* < 0.001). The laparoscopic surgery group had the highest proportion of urban insurance (82.68%), while the open surgery and TACE groups had the highest proportion of out-of-pocket payments (both over 21%). Furthermore, the distribution of patients with psychological anxiety differed among the treatment groups (*p* = 0.042).

**Table 1:** Patient baseline characteristics and treatment choice differences.

Variable	Total (*N* = 1928)	Open hepatectomy (*N* = 335)	Laparoscopic hepatectomy (*N* = 583)	Ablation (*N* = 126)	TACE (*N* = 881)	*p*-value
Age, years	57.96 ± 14.78	55.85 ± 13.86	57.33 ± 14.64	58.53 ± 14.92	57.96 ± 14.72	0.041
Male, *n* (%)	1,588 (82.36%)	275 (82.09%)	460 (78.90%)	92 (73.01%)	761 (86.38%)	<0.001
Payment, *n* (%)						<0.001
Urban insurance	1,460 (75.73%)	248 (74.03%)	482 (82.68%)	96 (76.19%)	634(71.96%)	
Government-funded	80 (4.15%)	10 (2.99%)	20 (3.43%)	5 (3.97%)	45 (5.11%)	
Rural cooperative	37 (1.92%)	7 (2.09%)	15 (2.57%)	2 (1.59%)	13 (1.48%)	
Out-of-pocket	351 (18.21%)	71 (21.19%)	66 (11.32%)	23 (18.25%)	191 (21.68%)	<0.001
Season of onset						0.125
Spring	468 (24.27%)	76 (22.69%)	124 (21.27%)	32 (25.40%)	236 (26.79%)	
Summer	520 (26.97%)	88 (26.27%)	171 (29.33%)	35 (27.78%)	226 (25.65%)	
Autumn	475 (24.64%)	99 (29.55%)	148 (25.39%)	24 (19.05%)	204 (23.16%)	
Winter	465 (24.12%)	73 (21.79%)	140 (24.01%)	35 (27.78%)	217 (24.63%)	
Psychological anxiety	162 (8.40%)	29 (8.66%)	43 (7.38%)	12 (9.52%)	78 (8.85%)	0.042
Metabolic diseases	676 (35.07%)	105 (31.34%)	204 (35.00%)	41 (32.54%)	326 (37.00%)	<0.001
Child-Pugh						<0.001
A	1,459 (75.67%)	229 (68.36%)	501 (85.93%)	82 (65.08%)	647 (73.44%)	
B	437 (22.67%)	99 (29.55%)	80 (13.72%)	40 (31.75%)	218 (24.74%)	
C	32 (1.66%)	8 (2.39%)	2 (0.34%)	4 (3.17%)	18 (2.04%)	
BCLC						<0.001
0–A	980 (50.83%)	90 (26.87%)	256 (43.91%)	77 (61.11%)	557 (63.22%)	
B	431 (22.35%)	96 (28.66%)	209 (35.85%)	17 (13.49%)	109 (12.37%)	
C	485 (25.16%)	142 (42.39%)	116 (19.90%)	28 (22.22%)	199 (22.59%)	
D	32 (1.66%)	8 (2.39%)	2 (0.34%)	3 (2.38%)	18 (2.04%)	
BMI						0.078
<18	99 (5.13%)	23 (6.87%)	19 (3.26%)	5 (3.97%)	52 (5.90%)	
18 ~ 24	1,160 (60.17%)	216 (64.48%)	341 (58.49%)	77 (61.11%)	526 (59.70%)	
>24	669 (34.70%)	97 (28.96%)	223 (38.25%)	44 (34.92%)	305 (34.62%)	

### 3.2 Treatment decision and guideline adherence and its influencing factors

**Figure 1:**
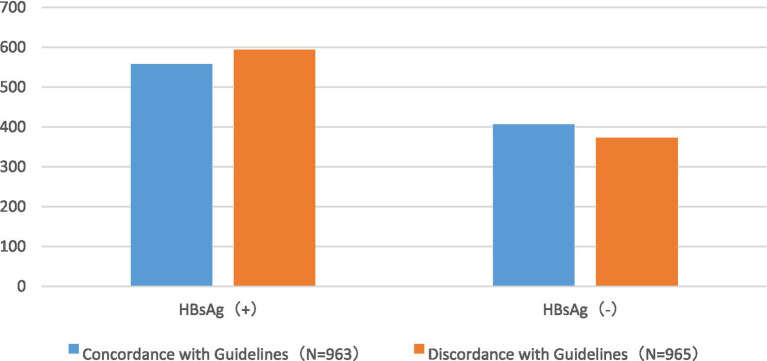
Comparison of guideline adherence by hepatitis B status.

**Figure 2:**
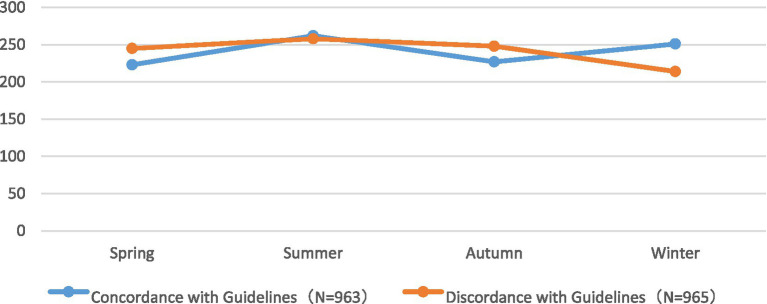
Comparison of guideline adherence across seasons.

**Figure 3:**
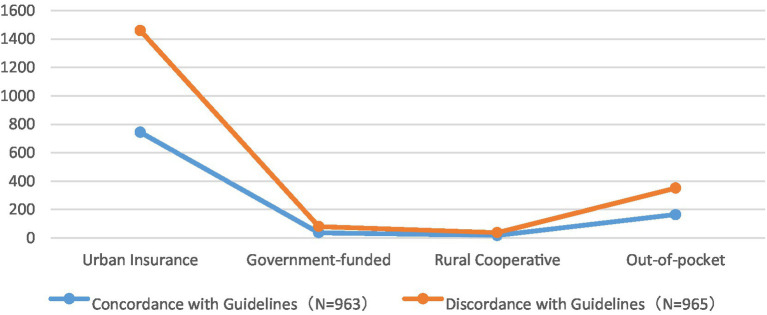
Comparison of guideline adherence by payment type.

**Figure 4:**
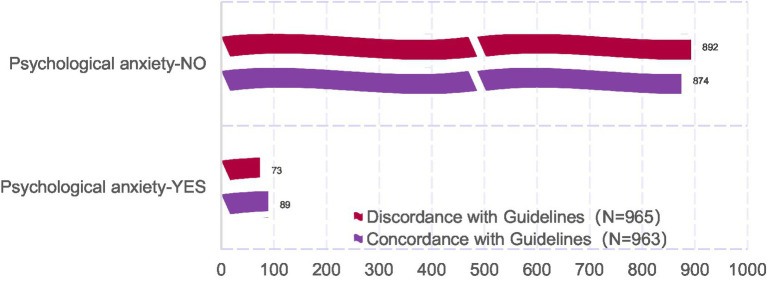
Comparison of guideline adherence by presence of psychological anxiety.

Among all 1928 patients, the initial treatment decision was guideline-adherent for 963 patients (49.95%) and non-guideline-adherent for 965 patients (50.05%). Subgroup analysis (Figures 1–4) revealed that payment method (*p* < 0.001) and psychological anxiety (*p* = 0.042) were significant factors influencing guideline adherence. Patients with urban insurance and those paying out-of-pocket had the highest decision deviation rates. Patients with anxiety had a significantly higher proportion of guideline deviation (9.1%) compared to those without anxiety (7.7%). HBV infection status, season of onset, and BMI group had no significant impact on guideline adherence.

### 3.3 Analysis of guideline adherence by BCLC stage

**Figure 5:**
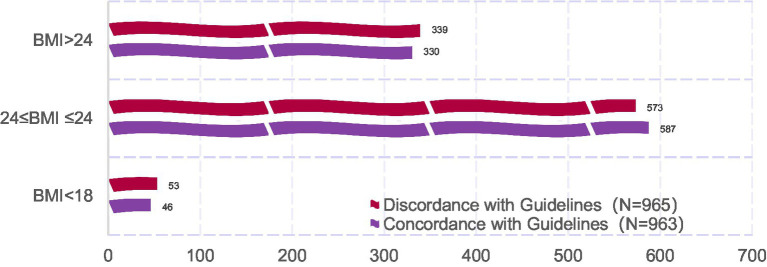
Comparison of guideline adherence across BMI groups.

**Figure 6:**
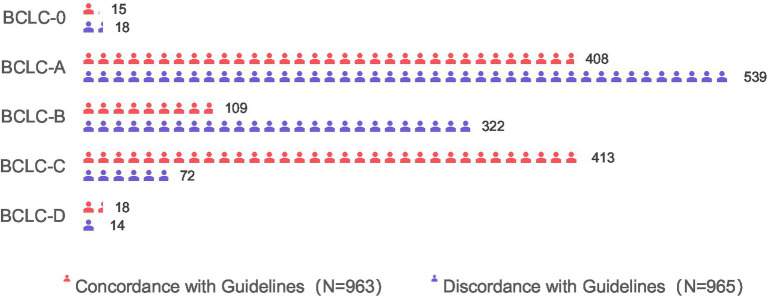
Comparison of guideline adherence across BCLC stages.

BCLC stage was the most critical factor affecting guideline adherence (*p* < 0.001). As shown in Figures 5, 6, Tables 2, 3: Early stage (BCLC 0–A): The proportion of early-stage patients in the non-guideline-adherent group was significantly higher at 57.7% (557/965), far exceeding the 43.9% (423/963) in the guideline-adherent group, suggesting substantial “under-treatment” or “over-treatment” in early-stage patients. Intermediate stage (BCLC-B): This was the group with the most severe deviation. 33.4% (322 patients) in the non-guideline-adherent group were BCLC-B patients, compared to only 11.3% (109 patients) in the guideline-adherent group, indicating a clear tendency towards “aggressive treatment.” Advanced stage (BCLC-C/D): Treatment decisions were highly guideline-adherent. Advanced-stage patients accounted for 44.8% (431 patients) in the guideline-adherent group, but only 8.9% (86 patients) in the non-guideline-adherent group.

**Table 2:** Comparison of guideline adherence by presence of psychological anxiety.

Psychological anxiety	Discordance with guidelines (*n*, %)	Concordance with guidelines (*n*, %)	Total
No	73 (7.6%)	892 (92.4%)	965
Yes	874 (92.7%)	89 (7.3%)	963
Total	947	981	1928

**Table 3:** Comparison of guideline adherence across BMI groups.

BMI group	Discordance with guidelines (*n*, %)	Concordance with Guidelines (*n*, %)	Total
BMI ≥ 24	339 (50.7%)	330 (49.3%)	669
18 ≤ BMI ≤ 24	573 (49.4%)	587 (50.6%)	1,160
BMI < 18	53 (53.5%)	46 (46.5%)	99
Total	965	963	1928

### 3.4 Multivariate regression analysis of survival outcomes and impact of treatment decision

**Figure 7:**
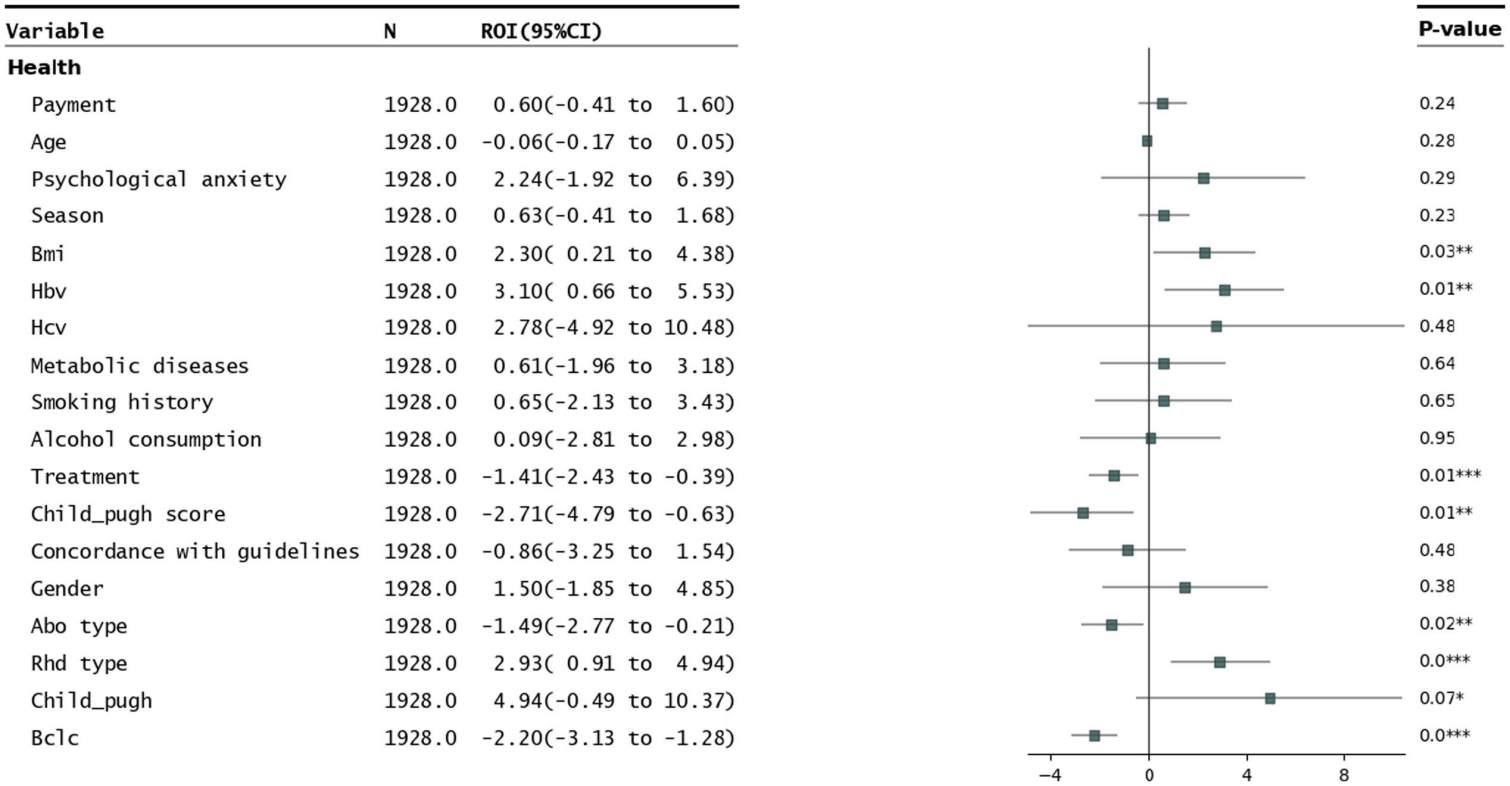
Multivariate COX regression analysis of survival outcomes.

**Figure 8:**
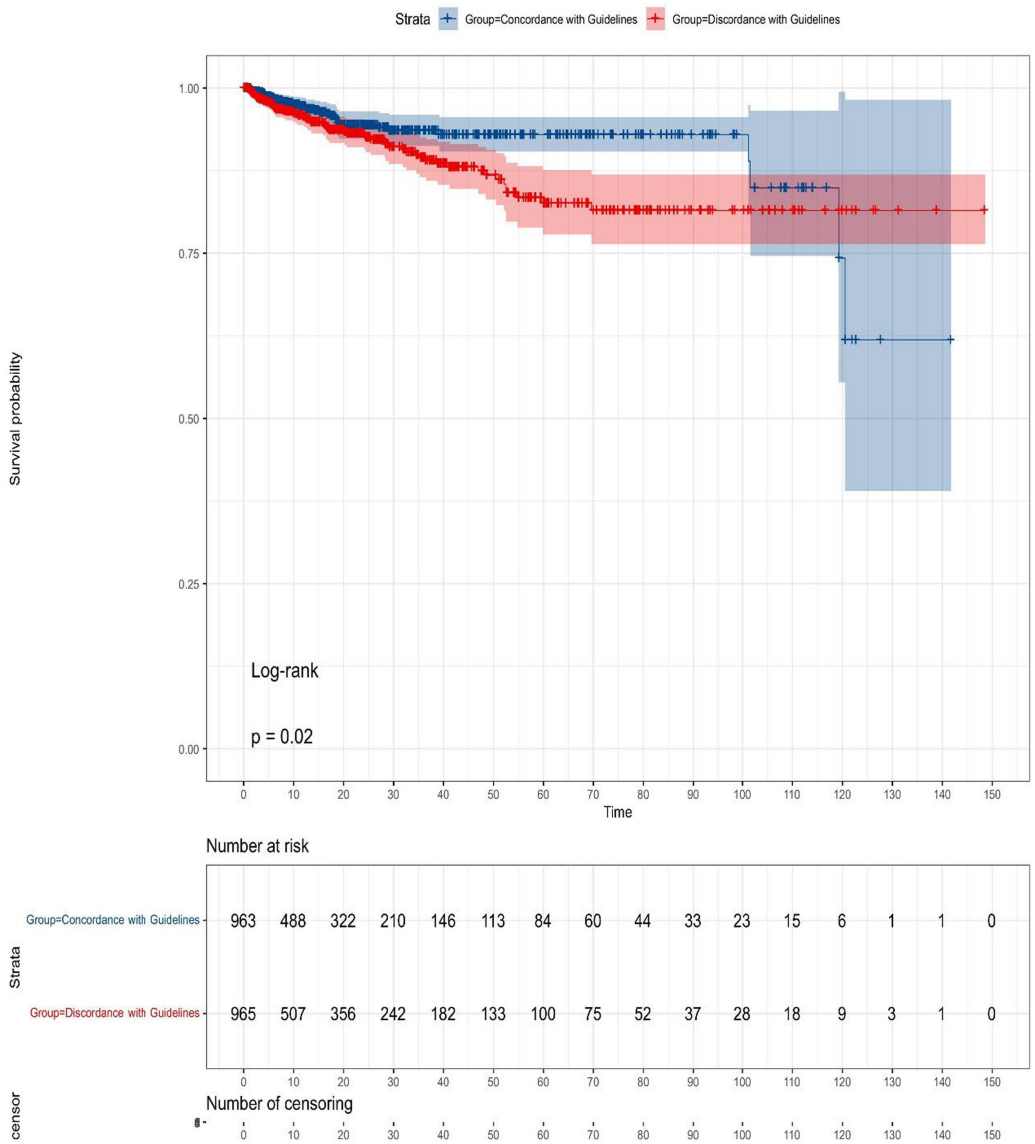
Impact of treatment decision on overall survival outcome in liver cancer.

**Figure 9:**
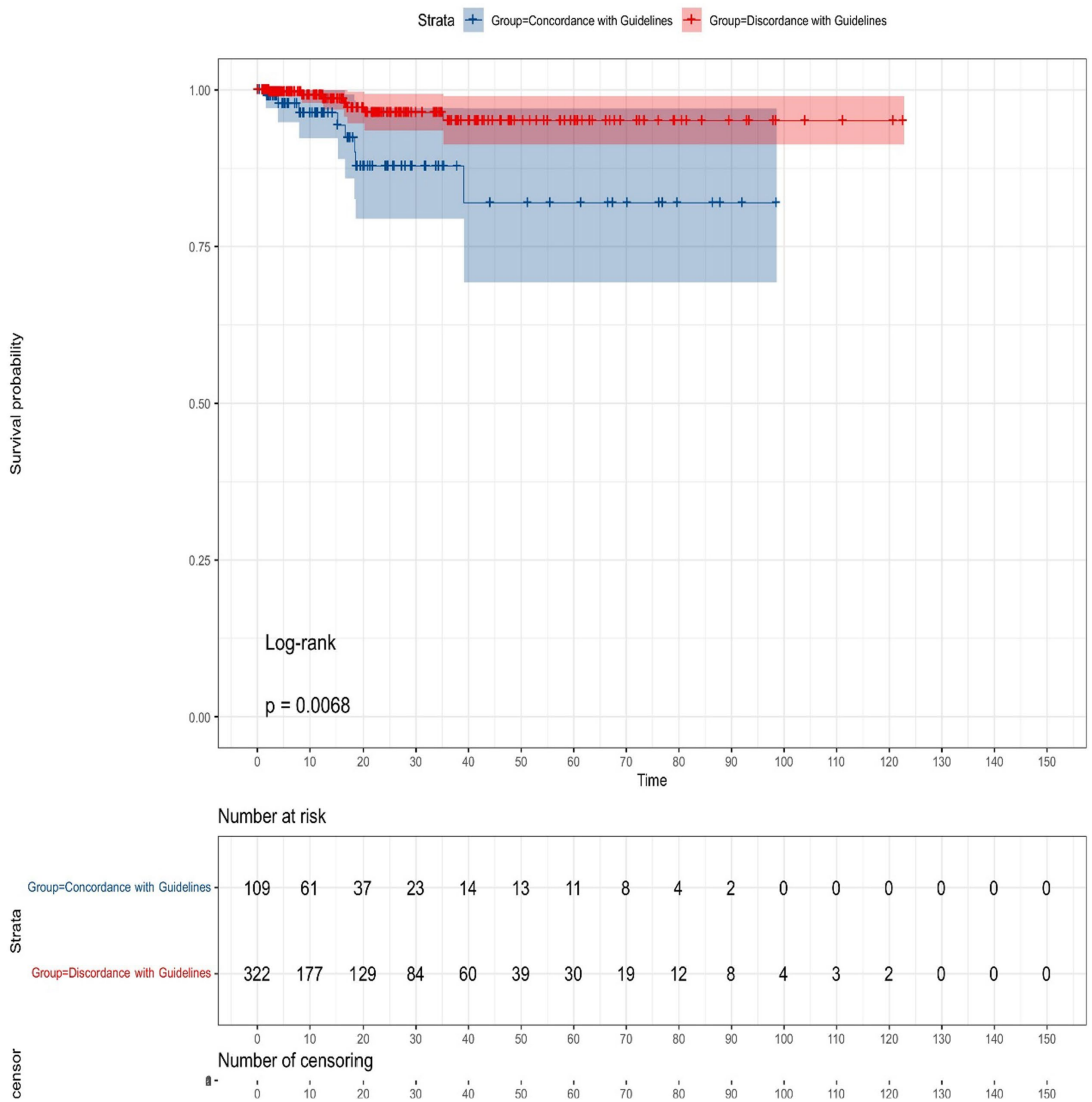
Impact of treatment decision on survival outcome in BCLC-B stage liver cancer.

Multivariate Cox regression analysis (Figure 7) showed that higher Child-Pugh grade, advanced BCLC stage, higher BMI, and HBV infection were independent risk factors for survival (HR > 1), while receiving treatment was a protective factor (HR < 1). Kaplan–Meier survival analysis (Figure 8) indicated that in the overall cohort, the guideline-adherent group had a better survival prognosis than the non-guideline-adherent group (*p* = 0.02). However, Figure 9 shows that among BCLC-B stage patients, the survival curve exhibited the opposite trend, with the non-guideline-adherent group having a longer median survival time than the strictly guideline-adherent group (*p* < 0.05). We additionally explored survival curves across other BCLC subgroups (stages 0–A and C–D) as part of a comprehensive analytical strategy. In these subgroups, the differences between guideline-adherent and non-adherent groups were minimal and showed no consistent survival separation. To maintain focus on the most representative and clinically meaningful findings, and to avoid potential visual selection bias, we present only the overall cohort and the BCLC-B subgroup where the most pronounced difference was observed. Explicitly, no attempt was made to selectively display only significant comparisons; instead, the decision to present these two survival curves reflects the underlying pattern of the data.

## 4 Discussion

This study, through large-sample retrospective data analysis, empirically examined the role of psychosocial factors in liver cancer treatment decisions and yielded several findings with important implications for clinical practice and medical education.

Firstly, this study confirms that treatment decision-making is a complex process that extends beyond pure clinical assessment. Although clinical factors such as Child-Pugh grade and BCLC stage remain dominant in decision-making, psychosocial variables like payment method and psychological anxiety were proven to be independent factors influencing treatment choice and guideline adherence. Economic burden may prevent patients from choosing the optimal treatment or force them to choose a suboptimal alternative, while anxiety may drive patients to avoid more traumatic curative surgeries in favor of treatments they perceive as “safer and more conservative”. This finding strongly supports the applicability of the biopsychosocial model in the field of liver cancer treatment (6), indicating that clinical guidelines alone cannot fully predict or explain real-world medical decision-making behavior (7).

Secondly, this study revealed a key and somewhat paradoxical phenomenon: among patients with BCLC-B stage (intermediate) liver cancer, treatment decisions that deviated from clinical guidelines were associated with better survival outcomes. This finding has profound significance. It may suggest that current population evidence-based clinical guidelines have limitations in addressing the high heterogeneity of intermediate-stage liver cancer (8). Some “off-protocol” intermediate-stage patients with good performance status, indolent tumor biology, or tumors confined to the liver might genuinely benefit from more aggressive local treatments such as surgical resection or ablation (9). This indicates that guidelines are “signposts” for clinical practice, not unbreakable “rules”. The value of the clinician lies in possessing sufficient expertise and clinical judgment to identify those specific individuals who might benefit from “deviating from the guidelines” (10).

The overall survival analysis showed that guideline adherence was still associated with a better overall survival trend, affirming the cornerstone role of guidelines in standardizing diagnosis and treatment and ensuring population prognosis (11). However, the finding in the intermediate-stage subgroup reminds us that “standardization” and “individualization” are not binary opposites but two dimensions that require careful weighing in modern clinical decision-making (12).

To further strengthen the transparency of our analytical process, we note that additional demographic and clinical variables beyond those formally retained were also explored through supplementary analyses. Specifically, marital status was assessed in the initial data extraction phase, given its known association with social support in previous oncology studies. However, due to the distributional characteristics of our cohort—where over 80% of patients were married and the unmarried group was too small for meaningful statistical comparison—this variable was not retained in the final model. Including it would risk Type II error and unreliable inference. Other variables such as area of residence and employer status were similarly examined but not retained due to lack of meaningful inferential value or significant predictive power in preliminary analyses. We explicitly acknowledge these analytical decisions here to further enhance the completeness and transparency of the study.

Finally, we address the limitations and potential selection bias. We acknowledge that the observed survival advantage in non-guideline-treated BCLC-B patients may be subject to residual confounding. Patients who received more aggressive “off-guideline” treatments (e.g., resection or ablation for BCLC-B disease) likely had better performance status, more favorable tumor biology (e.g., well-defined tumors, absence of vascular invasion), or better liver functional reserve compared with those who received guideline-concordant TACE. Future studies should employ propensity score matching or instrumental variable analysis to strengthen causal inference. Therefore, our finding should be interpreted as hypothesis-generating rather than definitive evidence that deviation from guidelines is superior in BCLC-B disease. Our study initially collected only two psychosocial variables—payment method (as a proxy for socioeconomic status) and documented psychological anxiety—for formal analysis. However, other potentially important factors, such as marital status, family influence, and educational level, were also collected at baseline. Due to the unique age structure of our cohort and the historical specificity of the corresponding cultural context, these factors did not significantly influence the outcomes in this study. Nevertheless, we believe that in the next 10–20 years, these factors will gradually exert distinct influences in the newly diagnosed HCC population, as demographic and cultural shifts occur.

## 5 Teaching implications

The results of this study provide clear directions for reform in the clinical training of liver cancer diagnosis and treatment, particularly in the cultivation of shared decision-making (SDM) skills among medical students and residents.

### 5.1 Restructuring teaching content

Traditional oncology teaching focuses on the mechanical correspondence between disease staging and treatment options. This study calls for adding a “decision science” module to the curriculum. Teaching content should shift from a “single correct answer” model towards demonstrating the complexity of clinical decisions, focusing on how to assess and integrate the patient’s psychosocial context (e.g., economic situation, emotional state, values and preferences) and combine it with clinical evidence to form the basis of decision-making.

### 5.2 Development of teaching cases

The data and cases from this study are excellent teaching materials. A series of structured teaching cases can be developed based on the real cases from this research. For example, designing a scenario for a BCLC-B stage patient with severe anxiety and economic difficulties, guiding students to discuss: How to communicate the pros and cons of different options with the patient? How to explain guideline recommendations without adding to their psychological burden? What social support resources can be mobilized? This trains core SDM skills—information delivery, preference exploration, and consensus building.

### 5.3 Elevating teaching objectives

The goal of medical education is not only to train doctors who know the guidelines well but also to cultivate clinicians with critical thinking and humanistic care. Teachers should guide students to recognize that their role is not only to provide treatment but also to act as guides and partners for patients, navigating the complex maze of decision-making together. The ultimate goal is to jointly select a plan that is medically sound, acceptable in the personal context, and emotionally bearable (13).

In summary, this study confirms that patient decisions deviating from guidelines are very common in medical scenarios, often driven by psychosocial factors. However, the most important implication of this study is to clarify that deviation from guidelines does not equate to an incorrect decision and may not preclude a better prognosis (14). In liver cancer treatment, especially in the highly heterogeneous early and intermediate stages, the essence of clinical decision-making is to find the optimal balance between population evidence and individual characteristics (15). Therefore, future clinical practice and medical education must move beyond simple compliance with guidelines and instead emphasize and train doctors to possess a more advanced ability (16): to conduct in-depth individualized consultation, decision-making, and guidance by integrating the patient’s specific tumor biology, liver function reserve, psychosocial background, and personal values, ultimately achieving truly patient-centered shared decision-making. This is both a science and an art, and it is the core competency that contemporary medical education must impart to future doctors.

## Data Availability

The raw data supporting the conclusions of this article will be made available by the authors, without undue reservation.
